# Supplementary material for: Potent and protective IGHV3-53/3-66 public antibodies and their shared escape mutant on the spike of SARS-CoV-2
Source: Nat Commun. 2021 Jul 9;12:4210. doi: 10.1038/s41467-021-24514-w (PMC8270942; doi:10.1038/s41467-021-24514-w)
Supplement: Supplementary file 1 — Supplementary information [file 41467_2021_24514_MOESM1_ESM.pdf]

Supplementary Information

Potent and protective IGHV3-53/3-66 public antibodies and their shared escape mutant on the spike of SARS-CoV-2

Qi Zhang[1,*], Bin Ju[2,*], Jiwan Ge[3,*], Jasper Fuk-Woo Chan[4,5,*], Lin Cheng[2,*], Ruoke Wang[1,*], Weijin Huang[6,*], Mengqi Fang[1], Peng Chen[1], Bing Zhou[2], Shuo Song[2], Sisi Shan[1], Baohua Yan[3], Senyan Zhang[3], Xiangyang Ge[2], Jiazhen Yu[2], Juanjuan Zhao[2,7], Haiyan Wang[2], Li Liu[5,8], Qining Lv[1], Lili Fu[1], Xuanling Shi[1], Kwok Yung Yuen[4,5], Lei Liu[2], Youchun Wang[6,#], Zhiwei Chen[4,5,8,#], Linqi Zhang[1,9,10,#], Xinquan Wang[3,#], Zheng Zhang[2,7,#]

[1]NexVac Research Center, Comprehensive AIDS Research Center, Beijing Advanced Innovation Center for Structural Biology, School of Medicine, Tsinghua University, Beijing 100084, China

[2]Institute for Hepatology, National Clinical Research Center for Infectious Disease, Shenzhen Third People's Hospital; The Second Affiliated Hospital, School of Medicine, Southern University of Science and Technology, Shenzhen 518112, Guangdong Province, China

[3]The Ministry of Education Key Laboratory of Protein Science, Beijing Advanced Innovation Center for Structural Biology, Beijing Frontier Research Center for Biological Structure, Collaborative Innovation Center for Biotherapy, School of Life Sciences, Tsinghua University, 100084 Beijing, China

[4]State Key Laboratory of Emerging Infectious Diseases, The University of Hong Kong, Pokfulam, Hong Kong SAR, People's Republic of China

[5]Department of Microbiology, Li Ka Shing Faculty of Medicine, The University of Hong Kong, Pokfulam, Hong Kong SAR, People's Republic of China

[6]Division of HIV/AIDS and Sex-Transmitted Virus Vaccines, National Institutes for Food and Drug Control, Beijing, China

[7]Shenzhen Bay Laboratory, Shenzhen 518055, Guangdong Province, China

[8]AIDS Institute, Li Ka Shing Faculty of Medicine, The University of Hong Kong, Pokfulam, Hong Kong Special Administrative Region, People's Republic of China

[9]Institute of Biopharmaceutical and Health Engineering, Tsinghua Shenzhen International Graduate School, Tsinghua University, Shenzhen 518055, China

[10]Institute of Biomedical Health Technology and Engineering, Shenzhen Bay Laboratory, Shenzhen 518132, China

*These authors contributed equally to this work.

#Correspondence: wangyc@nifdc.org.cn (Y.W.), zchenai@hku.hk (Z.C.), zhanglinqi@tsinghua.edu.cn (L.Z), xinquanwang@mail.tsinghua.edu.cn (X.W.), or zhangzheng1975@aliyun.com (Z.Z.)

Supplementary information

Supplementary Figures 1-6

Supplementary Tables 1-6

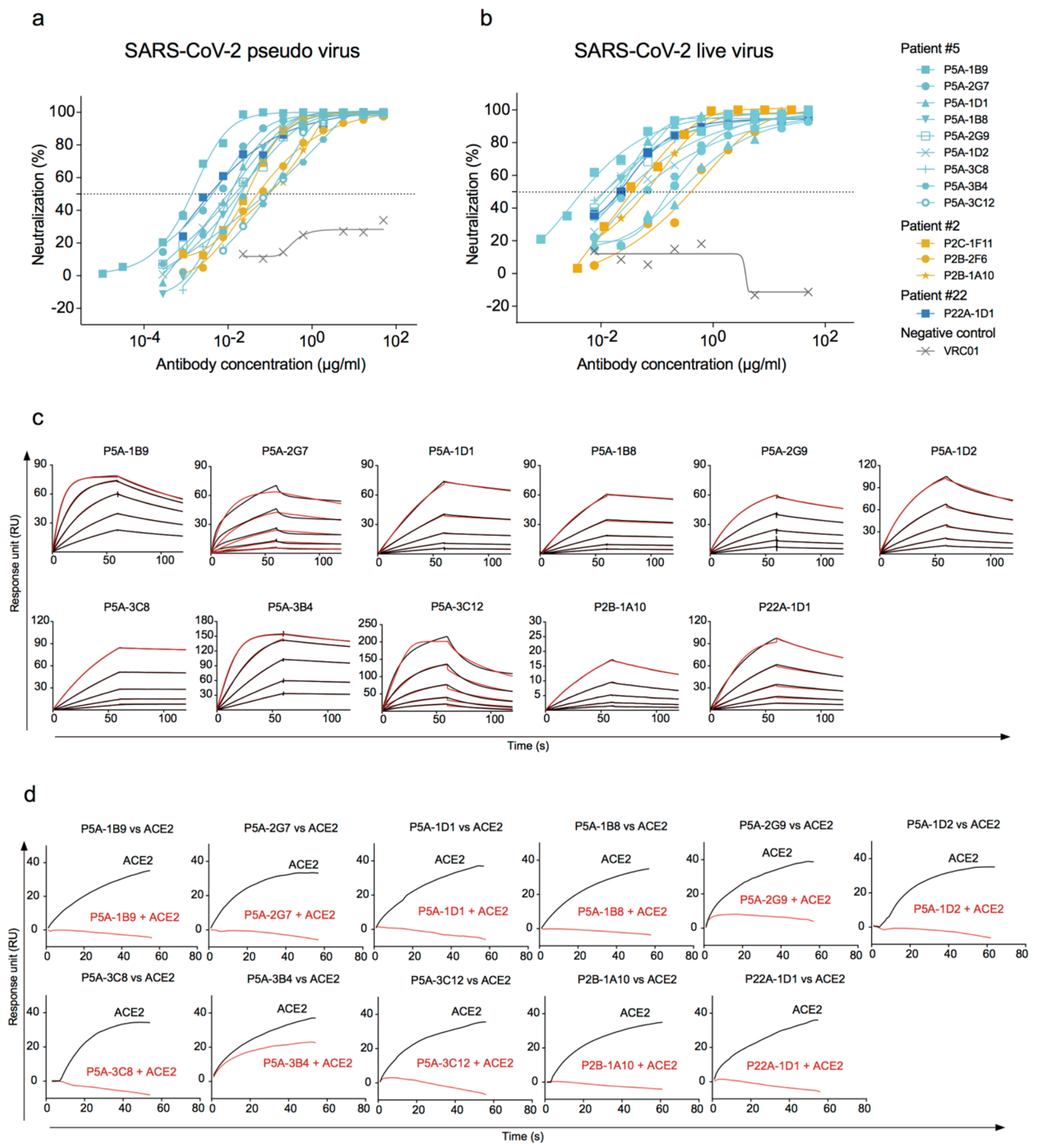

Supplementary Fig. 1 Neutralizing potency, binding kinetics, and ACE2 competition of top 13 antibodies.

Neutralizing activities of the top 13 antibodies against SARS-CoV-2 (**a**) pseudovirus or (**b**) live virus. VRC01 is an HIV-1 specific antibody and used here as a negative control. Results presented are representatives of two independent experiments. (**c**) The individual antibodies were captured on protein A covalently immobilized onto a CM5 sensor chip followed by injection of purified soluble SARS-CoV-2 WT RBD at five different concentrations. The black lines indicate the experimentally derived curves while the red lines represent fitted curves based on the experimental data. (**d**) The sensorgrams

show distinct binding patterns of ACE2 to SARS-CoV-2 RBD with (red curve) or without (black curve) prior incubation with each testing antibody. The competition capacity of each antibody is indicated by the level of reduction in response unit of ACE2 comparing with or without prior antibody incubation. Source data are provided as a Source Data file. Data for P2C-1F11 and P2B-2F6 have been published in the reference (Bin Ju, et al. Human neutralizing antibodies elicited by SARS-CoV-2 infection. Nature. 2020).

```
                                   HCDR1                          HCDR2
P5A-1D2   EVQLVESGGGLIQPGGSLRLSCAASGFIVSSNYMSWVRQAPGKGLEWVSIIYSGGSTYYADS
P22A-1D1  EVQLVESGGGLIQPGGSLRLSCAASGFTVSSNYMSWVRQAPGKGLEWVSVIYSGGSTYYADS
P5A-3C8   EVQLVESGGGLIQPGGSLRLSCAASGFTVSSNYMSWVRQAPGKGLEWVSFIYSGGSTYYADS
IGHV3-53  EVQLVESGGGLIQPGGSLRLSCAASGFTVSSNYMSWVRQAPGKGLEWVSVIYSGGSTYYADS

P2C-1F11  EVQLVESGGGLVQPGGSLRLSCAASGITVSSNYMNWVRQAPGKGLEWVSLIYSGGSTYYADS
IGHV3-66  EVQLVESGGGLVQPGGSLRLSCAASGFTVSSNYMSWVRQAPGKGLEWVSVIYSGGSTYYADS

                                                   HCDR3
P5A-1D2   VKGRFTISRDNSNNTLYLQMNSLRAEDTAVYYCARALQVGATSDYFDYWGQGTLVTVSS
P22A-1D1  VKGRFTISRDNSKNTLYLQMNSLRAEDTAVYYCARDRDYYG----MDVWGQGTTVTVSS
P5A-3C8   VKGRFTISRDNSKNTLYLQMNSLRAEDTAVYYCARDLQEHG----MDVWGQGTTVTVSS
IGHV3-53  VKGRFTISRDNSKNTLYLQMNSLRAEDTAVYYCAR------------------------

P2C-1F11  VKGRFTISRDNSKNTLYLQMNSLRAEDTAVYHCARDLVVYG----MDVWGQGTTVTVSS
IGHV3-66  VKGRFTISRDNSKNTLYLQMNSLRAEDTAVYHCAR------------------------
```

Supplementary Fig. 2 Multiple sequence alignment of the CDR1-CDR3 regions of the heavy chain sequences from the public clonetypes.

Included are antibodies P22A-1D1, P5A-1D2, P5A-3C8, and P2C-1F11 along with IGVH3-53/3-66, a top germline allele assignment for public antibodies shown. Bold letters show mutations from germline. Red letters show amino acids interacting with RBD.

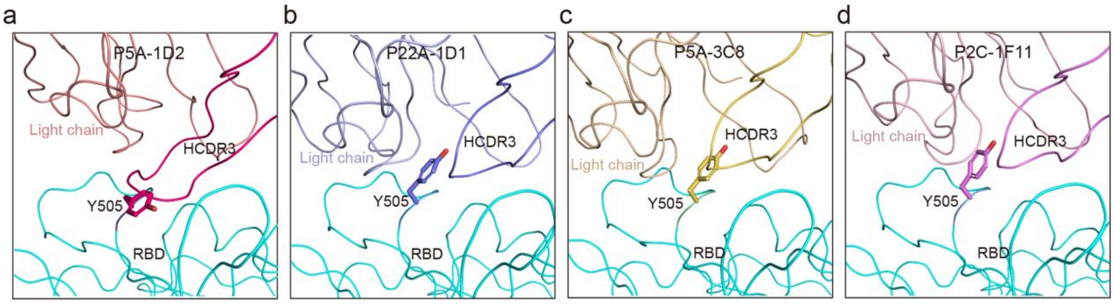

Supplementary Fig. 3 Y505 serves as an anchor residue for light chain binding.

For P5A-1D2 (**a**) Y505 displayed a unique conformation because of the binding of the long HCDR3, whereas for P22A-1D1 (**b**), P5A-3C8 (**c**) and P2C-1F11 (**d**), Y505 residue on RBD protruded into the wedge hole of the light chain.

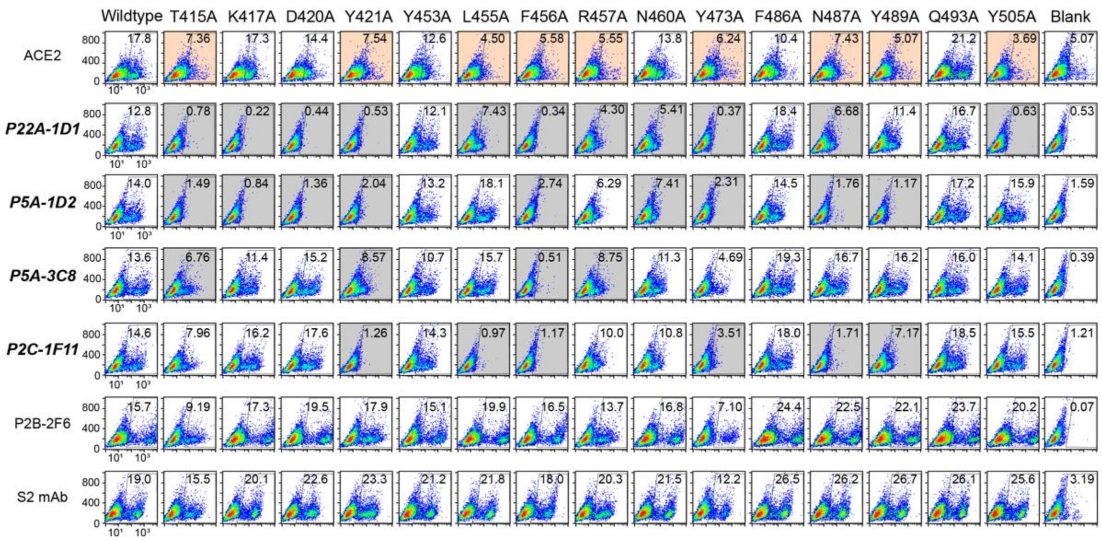

Supplementary Fig. 4 Impact of epitope mutations on binding of public antibodies.

Cell surface expressed wild type or mutant spike glycoprotein of SARS-CoV-2 were incubated with the ACE2, the public antibodies, and control antibodies, followed by staining with anti-human IgG Fc PE (for identified human antibodies), anti-mouse IgG FITC (for S2 mAb) or anti-his PE (for ACE2) secondary antibody and analyzed by FACS. P2B-2F6 recognizes a distinct epitope on SARS-CoV-2 RBD from those public antibodies and used here as positive control for the S1 protein of the spike. S2 is a mouse monoclonal antibody specific for S2 protein of the spike. The cell percentage in the gate were shown. Mutants that resulted in more than 80% reduction in binding are highlighted in either grey boxes for public antibodies or in orange boxes for ACE2. The percent reduction was determined by the MFI weighted by multiplying the number of positive cells in the selected gates and normalized in relative to that of wild type and S2 control. Data shown were from at least two independent experiments.

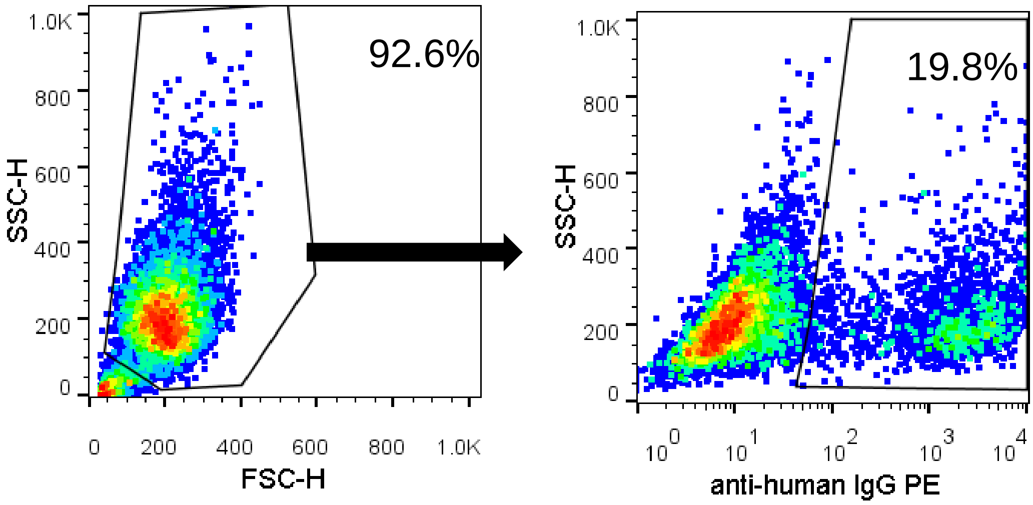

Supplementary Fig. 5 Gating strategy used for cell-surface staining analysis.

The gating strategy were used to study impact of epitope mutations on binding of public antibodies (Supplementary Fig. 4). HEK 293T cells transfected with plasmids encoding S or mutated S were incubated with ACE2 or antibodies, and stained with anti-his PE, anti-human IgG Fc PE or anti-mouse IgG FITC.

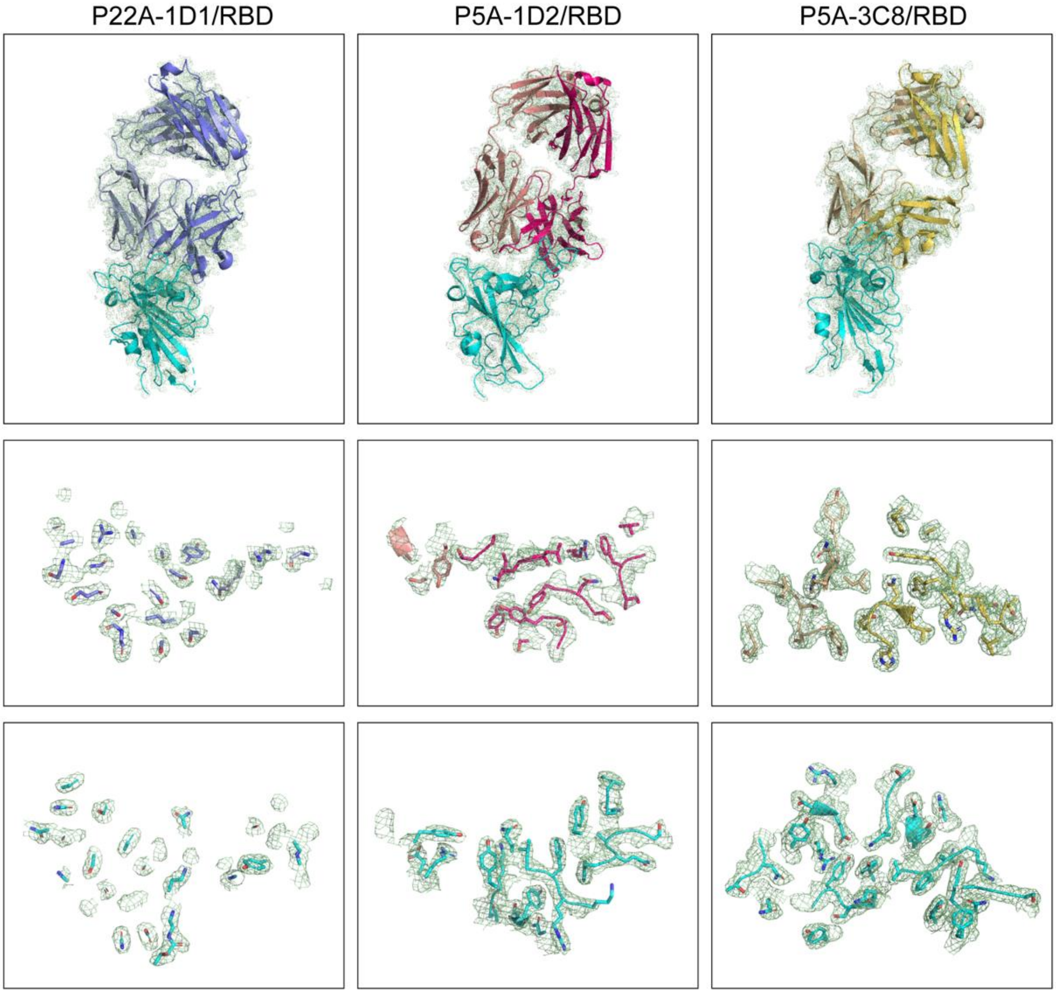

Supplementary Fig. 6 2Fo − Fc electron density maps of P5A-1D2, P5A-3C8 and P22A-1D1/SARS-CoV-2 RBD complexes.

2Fo − Fc electron density maps contoured at 1.0σ at the binding interface between the SARS-CoV-2 RBD and antibodies were shown. Upper: the whole structure; Middle: the part of antibodies in the binding interface; Lower: the part of RBD in the binding interface.

Supplementary Table 1. The IC_{50} of analyzed 165 antibodies.

mAbs	Pseudovirus IC_{50} (μg/ml)	Pseudovirus IC_{80} (μg/ml)	Live virus IC_{50} (μg/ml)	Live virus IC_{80} (μg/ml)
P5A-1B9	0.0014	0.0053	0.0043	0.0441
P22A-1D1	0.0038	0.0625	0.0198	0.1321
P5A-2G7	0.0044	0.0287	0.1814	0.8355
P5A-1D1	0.0096	0.0691	0.0189	0.0743
P5A-1B8	0.0115	0.0501	0.0168	0.0857
P5A-2G9	0.0158	0.1466	0.0113	0.1187
P5A-1D2	0.0186	0.1025	0.0273	0.4325
P5A-3C8	0.0206	0.1031	0.0112	0.1499
P2C-1F11*	0.0286	0.1195	0.0323	0.1779
P2B-2F6*	0.0500	0.6074	0.4074	2.4309
P2B-1A10	0.0974	0.7446	0.0639	0.3053
P5A-3B4	0.0993	1.0657	0.0561	1.0080
P5A-3C12	0.0996	0.4679	0.2636	2.6783
P2B-1G5	0.1100	0.3700	0.0302	0.1725
P5A-1C8	0.1162	0.4621	0.1553	1.3370
P4A-1H6	0.1370	0.7670	0.0722	2.0307
P5A-3A6	0.2340	1.2670	0.4443	18.3749
P5A-1B6	0.2528	1.3719	0.8932	5.9133
P5A-2D11	0.3889	1.4758	0.1154	4.1504
P5A-2H3	0.5042	2.0522	0.1214	0.7471
P2C-1A3*	0.6200	5.9400	0.2827	1.4587
P5A-2F11	0.6300	1.9400	0.4942	6.9416
P2B-1A1	0.6900	2.4100	0.2218	2.1498
P5A-3A1	0.9231	4.2357	0.6713	26.2193
P22A-1D8	0.9889	6.1038	n.d.	n.d.
P8A-1D5	1.0550	5.8355	n.d.	n.d.
P5A-2G5	1.1528	5.6968	n.d.	n.d.
P4B-1E12	1.3813	14.9370	n.d.	n.d.
P5A-2D6	1.4600	15.1300	n.d.	n.d.
P4A-2D9	1.5400	5.9600	n.d.	n.d.
P2C-1C10*	2.6200	4.6400	11.1204	>50
P5A-2G12	2.6540	12.1251	n.d.	n.d.
P2B-1D9	3.1200	6.4200	n.d.	n.d.
P5A-3A7	3.2500	>50	n.d.	n.d.
P4B-1F4	3.4486	9.0132	n.d.	n.d.
P2B-1G1	4.2200	11.6200	n.d.	n.d.
P16A-1A3	4.5554	15.9269	n.d.	n.d.
P1A-1C2	5.0337	21.4613	n.d.	n.d.
P2B-2G4*	5.1100	>50	2.9005	47.7043
P5A-1D10	5.7200	28.8700	n.d.	n.d.
P2B-1E4	5.9600	16.9200	n.d.	n.d.
P5A-2E1	6.0300	8.7600	n.d.	n.d.
P5A-2C9	6.4000	>50	n.d.	n.d.
P5A-2D10	6.5647	19.6532	n.d.	n.d.
P2B-1F11	6.5900	14.4100	n.d.	n.d.
P2B-1A12	7.2200	>50	n.d.	n.d.

P5A-2C12	7.3500	>50	n.d.	n.d.
P2A-1A8*	7.6800	26.4100	35.8664	>50
P5A-3D12	7.9600	>50	n.d.	n.d.
P5A-1B1	8.0100	40.4900	n.d.	n.d.
P2A-1A10*	8.5700	39.4400	1.6395	22.1536
P2B-2G9	8.8900	>50	n.d.	n.d.
P2B-1F10	9.7400	49.7800	n.d.	n.d.
P2B-1F5	10.3000	>50	n.d.	n.d.
P2C-1D5*	10.6500	25.3600	n.d.	n.d.
P2B-2F11	13.1100	>50	n.d.	n.d.
P16A-1B3	13.8943	>50	n.d.	n.d.
P5A-2E5	14.0700	>50	n.d.	n.d.
P2C-1F4	15.9600	>50	n.d.	n.d.
P2A-1B3*	16.7700	>50	n.d.	n.d.
P5A-1D6	18.2000	>50	n.d.	n.d.
P5A-2E12	20.4745	>50	n.d.	n.d.
P5A-1A1	23.8500	>50	n.d.	n.d.
P22A-1D7	23.8734	>50	n.d.	n.d.
P2A-1A9*	26.2700	>50	n.d.	n.d.
P16A-1A8	33.6854	>50	n.d.	n.d.
P2C-1C8*	34.3800	>50	n.d.	n.d.
P2B-2G11*	34.8400	>50	n.d.	n.d.
P2B-1B4	35.3200	>50	n.d.	n.d.
P4A-2E10	35.3500	>50	n.d.	n.d.
P5A-3C3	36.1300	>50	n.d.	n.d.
P1A-1C10*	>50	>50	n.d.	n.d.
P1A-1C7*	>50	>50	n.d.	n.d.
P1A-1D1*	>50	>50	n.d.	n.d.
P2C-1E1*	>50	>50	n.d.	n.d.
P2C-1B12	>50	>50	n.d.	n.d.
P2C-1E5	>50	>50	n.d.	n.d.
P2B-1G8	>50	>50	n.d.	n.d.
P4A-2A2	>50	>50	n.d.	n.d.
P2C-1A7	>50	>50	n.d.	n.d.
P5A-1A12	>50	>50	n.d.	n.d.
P5A-1B10	>50	>50	n.d.	n.d.
P5A-1C9	>50	>50	n.d.	n.d.
P5A-1C10	>50	>50	n.d.	n.d.
P5A-1C11	>50	>50	n.d.	n.d.
P4A-2C1	>50	>50	n.d.	n.d.
P2B-1D6	>50	>50	n.d.	n.d.
P2B-1E12	>50	>50	n.d.	n.d.
P2A-1B10	>50	>50	n.d.	n.d.
P5A-2C8	>50	>50	n.d.	n.d.
P5A-3B8	>50	>50	n.d.	n.d.
P5A-3C10	>50	>50	n.d.	n.d.
P5A-3B9	>50	>50	n.d.	n.d.
P5A-1C4	>50	>50	n.d.	n.d.
P5A-2D3	>50	>50	n.d.	n.d.
P5A-1D8	>50	>50	n.d.	n.d.

P5A-2G11	>50	>50	n.d.	n.d.
P5A-2D12	>50	>50	n.d.	n.d.
P5A-2E8	>50	>50	n.d.	n.d.
P5A-3A2	>50	>50	n.d.	n.d.
P5A-1A5	>50	>50	n.d.	n.d.
P5A-3A10	>50	>50	n.d.	n.d.
P5A-2D7	>50	>50	n.d.	n.d.
P5A-3D9	>50	>50	n.d.	n.d.
P5A-3C1	>50	>50	n.d.	n.d.
P5A-2G4	>50	>50	n.d.	n.d.
P3A-1F1	>50	>50	n.d.	n.d.
P4B-1E7	>50	>50	n.d.	n.d.
P16A-1B12	>50	>50	n.d.	n.d.
P22A-1E10	>50	>50	n.d.	n.d.
P5A-3A11	>50	>50	n.d.	n.d.
P5A-1B11	>50	>50	n.d.	n.d.
P5A-2E9	>50	>50	n.d.	n.d.
P5A-1A2	>50	>50	n.d.	n.d.
P5A-1B12	>50	>50	n.d.	n.d.
P5A-2C7	>50	>50	n.d.	n.d.
P5A-2F7	>50	>50	n.d.	n.d.
P5A-2F9	>50	>50	n.d.	n.d.
P5A-3B10	>50	>50	n.d.	n.d.
P5A-1C6	>50	>50	n.d.	n.d.
P5A-2C10	>50	>50	n.d.	n.d.
P5A-2D5	>50	>50	n.d.	n.d.
P5A-2F1	>50	>50	n.d.	n.d.
P5A-2G8	>50	>50	n.d.	n.d.
P1A-1C6	>50	>50	n.d.	n.d.
P1A-1D3	>50	>50	n.d.	n.d.
P1A-1D5	>50	>50	n.d.	n.d.
P2C-1A6	>50	>50	n.d.	n.d.
P3A-1G8	>50	>50	n.d.	n.d.
P4A-2A10	>50	>50	n.d.	n.d.
P4B-1F6	>50	>50	n.d.	n.d.
P4B-1E11	>50	>50	n.d.	n.d.
P4A-2A8	>50	>50	n.d.	n.d.
P4A-1H5	>50	>50	n.d.	n.d.
P4A-2B3	>50	>50	n.d.	n.d.
P4B-1G5	>50	>50	n.d.	n.d.
P4B-1F10	>50	>50	n.d.	n.d.
P4A-2D1	>50	>50	n.d.	n.d.
P4A-2D2	>50	>50	n.d.	n.d.
P4A-2C12	>50	>50	n.d.	n.d.
P8A-1A8	>50	>50	n.d.	n.d.
P8A-1C6	>50	>50	n.d.	n.d.
P8A-1A5	>50	>50	n.d.	n.d.
P16A-1B5	>50	>50	n.d.	n.d.
P16A-1C6	>50	>50	n.d.	n.d.
P16A-1C1	>50	>50	n.d.	n.d.

P16A-1A5	>50	>50	n.d.	n.d.
P16A-1A12	>50	>50	n.d.	n.d.
P16A-1B1	>50	>50	n.d.	n.d.
P16A-1B8	>50	>50	n.d.	n.d.
P16A-1A7	>50	>50	n.d.	n.d.
P16A-1A10	>50	>50	n.d.	n.d.
P22A-1D2	>50	>50	n.d.	n.d.
P22A-1D5	>50	>50	n.d.	n.d.
P22A-1E6	>50	>50	n.d.	n.d.
P1A-1D6	>50	>50	n.d.	n.d.
P2B-2G10	>50	>50	n.d.	n.d.
P2B-1C3	>50	>50	n.d.	n.d.
P2B-1D11	>50	>50	n.d.	n.d.
P2B-1E2	>50	>50	n.d.	n.d.
P2B-1F9	>50	>50	n.d.	n.d.
P22A-1E8	>50	>50	n.d.	n.d.
P2C-1D7	n.d.	n.d.	n.d.	n.d.
P1A-1B2*	n.d.	n.d.	n.d.	n.d.
P1A-1C1*	n.d.	n.d.	n.d.	n.d.

*Published in the reference (Bin Ju, et al. Human neutralizing antibodies elicited by SARS-CoV-2 infection. Nature. 2020).

n.d.: not determined.

Supplementary Table 2. Data collection and refinement statistics (molecular replacement).

**Data collection**			
	**SARS-CoV-2 RBD-P22A-1D1 complex**	**SARS-CoV-2 RBD-P5A-1D2 complex**	**SARS-CoV-2 RBD-P5A-3C8 complex**
Wavelength (Å)	0.97918	0.97918	0.97918
Resolution range (Å)	50.00-2.40 (2.46-2.40) *	50.00-2.56 (2.66-2.56) *	68.22-2.36 (2.48-2.36) *
Space group	*C*2	*C*2	$P\,2_1\,2_1\,2$
Unit cell dimensions			
a, *b*, *c* (Å)	193.34, 86.60, 57.16	158.75, 67.51, 154.37	112.54, 171.57, 54.87
α, β, γ (°)	90, 99.25, 90	90, 112.18, 90	90, 90, 90
Unique reflections	36392 (3573)	47159 (3117)	43632 (4424)
Completeness (%)	99.73 (98.48)	95.9 (64.3)	97.3 (100.0)
Mean I/sigma (I)	13.1 (1.9)	9.5 (1.0)	13.0 (2.4)
Redundancy	6.7 (7.0)	6.3 (3.7)	13.2 (13.8)
R_{merge} (%)	11.4 (98.3)	11.7 (79.9)	13.7 (115.7)
R_{pim} (%)	6.2 (42.7)	7.0 (41.2)	5.6 (46.4)
CC1/2	0.989 (0.655)	0.989 (0.450)	0.997 (0.808)
Wilson B-factor (Å^2)	37.35	51.17	45.42
**Structure refinement**			
Resolution (Å)	47.71-2.40	36.52-2.56	50.98-2.36
R_{work} /R_{free} (%)	19.4/22.4	20.3/24.7	19.4/21.8
No. atoms			
Protein	4713	9446	4770
Glycan	14	28	14
Protein residues	614	1249	623
B-factors (Å^2)			
Protein	42.00	53.89	47.25
Glycan	69.13	115.29	52.72
RMSD			
Bonds length (Å)	0.014	0.008	0.009
Bonds angles (°)	1.39	1.07	1.26
Ramachandran plot			
Favored (%)	96.52	94.39	96.58
Allowed (%)	3.32	5.36	2.93
Outliers (%)	0.17	0.24	0.49

*One crystal for the data

*Values in parentheses are for highest-resolution shell.

Supplementary Table 3. Comparison of buried surface area of light chain IGKV1-9 and IGKV3-20 usage among public antibodies.

	P5A-1D2		P5A-3C8		P22A-1D1		P2C-1F11		B38		CB6		CC12.1		CC12.3		C105		CV30	
	H	L	H	L	H	L	H	L	H	L	H	L	H	L	H	L	H	L	H	L
CDR3	IGHV3-53	IGLV1-40	IGHV3-53	IGKV1-9	IGHV3-53	IGKV1-9	IGHV3-66	IGKV3-20	IGHV3-53	IGKV1-9	IGHV3-66	IGKV1-39	IGHV3-53	IGKV1-9	IGHV3-53	IGKV3-20	IGHV3-53	IGLV2-8	IGHV3-53	IGKV3-20
Buried surface (Å^2)	839.6	164.0	725.2	547.9	728.7	408.8	774.8	204.3	736.3	486.0	732.6	355.4	786.3	560.2	724.0	167.0	677.7	266.2	791.5	247.6
Total (Å^2)	1003.6		1273.1		1137.5		979.1		1222.3		1088.0		1346.5		891.0		943.9		1039.1	

Supplementary Table 4. Contacts between SARS-CoV-2 RBD and P22A-1D1, P5A-1D2, P5A-3C8, P2C-1F11 (distance cutoff 4Å).

RBD/P22A-1D1				RBD/P5A-1D2				RBD/P5A-3C8				RBD/P2C-1F11			
Heavy chain	RBD	Light chain	RBD	Heavy chain	RBD	Light chain	RBD	Heavy chain	RBD	Light chain	RBD	Heavy chain	RBD	Light chain	RBD
G26	F486, N487	Q27	G502, V503	G26	G476, S477	A31	Y505	G26	N487	G28	G502	G26	N487	S28	G502, Y505
F27	N487	G28	T500, N501, G502, Y505	F27	A475, N487	Y33	R403, D405	F27	A475, N487	I29	N501, G502, Y505	I27	A475, N487	V29	Y505
T28	G476, S477	I29	Y505	I28	A475, G476, S477	S95	D405	T28	A475, G476, S477	S30	N501, Y505	T28	A475, G476, S477	S30	Y505
S31	K458, Y473	S30	G496, N501	S31	Y473, Q474			S31	K458, Y473	S31	G496, Q498, N501	S31	K458, Y473, Q474	Y33	R403, Y453
N32	A475	Y32	R403, Y453, Q493, Y495, Y505	N32	A475			N32	A475	Y32	Y449, Q493, S494, Y495	N32	A475	Q91	Y505
Y33	K417, L455, F456	S67	Q498	Y33	K417, Y421, L455, F456			Y33	K417, Y421, L455	S67	T500	Y33	Y421, L455, F456	Y92	Y505
Y52	G416, K417, D420, Y421	H90	Y505	Y52	K417, D420, Y421			Y52	G416, K417, D420, Y421	G68	T500	Y52	G416, K417, D420, Y421		
S53	Y421, R457, K458	L91	Y505	S53	Y421, R457, K458, Y473			S53	Y421, R457, K458, Y473	H90	Y505	S53	Y421, R457, K458, Y473		
G54	K458, N460	N92	R403, K417	G54	Y421, K458, N460			G54	Y421, R457, K458, N460	L91	K417	G54	Y421, K458, N460		

S56	T415, D420	Y94	Y505	S56	T415, D420, N460			S56	T415, D420	N92	Y505	S56	T415, D420		
Y58	T415, G416			Y58	T415, G416			Y58	T415, G416	S93	D405, E406, Y505	Y58	T415, G416		
R97	F486, N487, Y489			R87	N487			R97	F486, N487, Y489	Y94	R408	R97	A475, F486, N487, Y489		
R99	F456, Y489			R97	A475, Y489			L99	F456, Y489			L99	F456, Y489		
D100	K417, L455			L99	F456, Y489			Q100	K417, L455			V101	L455		
Y101	L455, Q493			Q100	K417, Y453, L455, Q493			E101	K417, Y453, L455, Q493			Y102	Q493		
Y102	L455, F456, Q493			V101	F456, Q493			H102	Q493			D105	F486		
D105	F486			G102	Q493										
				A103	Q493										
				T104	Y505										
				D106	K417										

Supplementary Table 5. Interactions between public antibodies and SARS-CoV-2 K417 residue.

	RBD	antibody	Length (Å)	Interaction	Total
P2C-1F11	E/K417/N[N]	H/Y52/CE2[C]	3.84		7
		H/Y52/OH[O]	3.87	Hydrogen bond	
	E/K417/CG[C]	H/Y52/CE2[C]	3.85		
		H/Y52/CZ[C]	3.97		
		H/Y52/OH[O]	3.32		
	E/K417/CE[C]	H/Y52/OH[O]	3.69		
	E/K417/NZ[N]	H/Y52/OH[O]	3.19	Hydrogen bond	
P22A-1D1	E/K417/N[N]	H/Y52/CE2[C]	3.76		11
	E/K417/CG[C]	H/Y52/CE2[C]	3.65		
		H/Y33/OH[O]	3.53		
		H/Y52/OH[O]	3.87		
	E/K417/CD[C]	H/Y33/OH[O]	3.48		
		H/D100/OD2[O]	3.85		
	E/K417/CE[C]	H/D100/OD1[O]	3.94		
		H/D100/OD2[O]	3.71		
	E/K417/NZ[N]	H/D100/CG[C]	3.72		
		H/D100/OD1[O]	3.83	Salt bridge	
		H/D100/OD2[O]	2.87	Salt bridge	
P5A-1D2	E/K417/CG[C]	H/Y33/OH[O]	3.74		13
		H/Y52/CE2[C]	3.65		
	E/K417/CD[C]	H/Q100/OE1[O]	3.57		
	E/K417/CE[C]	H/Q100/OE1[O]	3.59		
		H/D106/OD1[O]	3.74		
		H/Y52/OH[O]	3.76		
	E/K417/NZ[N]	H/Q100/CB[C]	3.99		
		H/Q100/CG[C]	3.83		
		H/Q100/CD[C]	3.55		
		H/Q100/OE1[O]	2.61	Hydrogen bond	
		H/D106/CG[C]	3.32		
		H/D106/OD1[O]	2.70	Salt bridge	
		H/D106/OD2[O]	3.21	Salt bridge	
P5A-3C8	E/K417/N[N]	H/Y52/CE2[C]	3.73		11
	E/K417/CG[C]	H/Y33/OH[O]	3.18		
		H/Y52/CE2[C]	3.90		
	E/K417/CD[C]	H/Y33/OH[O]	3.66		
		H/Q100/OE1[O]	3.29		
	E/K417/CE[C]	H/Q100/OE1[O]	3.59		
	E/K417/NZ[N]	H/E101/OE2[O]	3.96	Salt bridge	
		H/E101/OE1[O]	3.99	Salt bridge	
		H/Q100/CD[C]	3.90		
		H/Q100/OE1[O]	2.82	Hydrogen bond	
		L/L91/CD2[C]	3.78		

Supplementary Table 6. The list of primers.

Primer name	Sequence (5'-3')
VSV-SARS2-cDNA-RBD-F	CACGTGTGATCAGATATCGCGGCCGCACCAATCTGTGCCCTTTCGG
VSV-SARS2-cDNA-RBD-R	GAAGGCACAGCAGATCTGGATCCCTTAGGGCCGCACACGGTA
Rdrp-F	CGCATACAGTCTTRCAGGCT
Rdrp-R	GTGTGATGTTGAWATGACATGGTC
sgmRNA-F-44	CGATCTCTTGTAGATCTGTTCTC
sgmRNA-R-N-28458	TCCTTGCCATGTTGAGTGAG
